# CTLA-4 promotes lymphoma progression through tumor stem cell enrichment and immunosuppression

**DOI:** 10.1515/biol-2021-0094

**Published:** 2021-09-06

**Authors:** Yan Chen, Meng Li, Jian Cao, Guohong Cai, Xiantao Li, Yuejiao Liu, Wen Chen

**Affiliations:** Department of Integrated TCM and Western Medicine, Central Hospital of Yiyang City, Yiyang, Hunan, 413200, China; Department of Pathology, The 8th Medical Center, Chinese PLA General Hospital, Beijing 100091, China

**Keywords:** lymphoma, CTLA-4, regulatory T cell, tumor stem cell

## Abstract

The recurrence rate of lymphoma is very high, and tumor stem cells may be an important mechanism. Cytotoxic T lymphocyte-associated antigen 4 (CTLA-4) can inhibit antitumor immunity and promote cancer progression, but its role and mechanism in lymphoma are still unclear. Here we collected lymphoma tissue and peripheral blood from patients with diffuse large B-cell lymphoma (DLBCL). Results showed that CTLA-4 expression and CD44+ cell in the high-risk group were significantly higher than that in the low-risk group. Correlation analysis showed that CTLA-4 expression positively correlated with CD44+ cell in lymphoma tissue and regulatory T (Treg) cells in lymphocytes. *In vitro* experiment showed that CTLA-4 increased the ratio of lymphoma stem cells, and proliferation and invasion of lymphoma cells through TGF-β pathway. Moreover, CTLA-4 enhanced the proliferation of Treg cells induced by lymphoma cells. Animal experiments showed that CTLA-4 can promote transplanted lymphoma growth. Immunohistochemistry results showed that both Ki-67 and CD44+ cells increased significantly in the CTLA-4 group. TGF-β neutralization can significantly block these effects of CTLA-4. In conclusion, CTLA-4 promoted DLBCL progression through lymphoma stem cell enrichment and immunosuppression.

## 1 Introduction

Diffuse large B-cell lymphoma (DLBCL) is the most common type of lymphoma. The symptoms of lymphoma are not clinically specific, so early diagnosis is very difficult [1]. Many patients are in advanced stages when they are diagnosed. Although chemotherapy and radiotherapy can achieve temporary remission, the recurrence rate of lymphoma is very high [2]. Clinical research showed that over 40% of DLBCL will recur in months to years after treatment, and it was often difficult to cure after recurrence [3]. Therefore, it is of great clinical significance to find new targets for the diagnosis and treatment of lymphoma.

Many studies have proved that there are tumor stem cells in lymphoma [4,5]. Tumor stem cells are highly resistant to radiotherapy and chemotherapy, which is an important mechanism of lymphoma recurrence and metastasis [6]. CD44 is one of the important markers for tumor stem cells. CD44+ cells generally express Bmi-1 strongly and have a high level of cell proliferation [7]. Immune escape is another important cause of lymphoma recurrence caused by tumor stem cells. In lymphoma, NK cell activity persistently decreases, compared to controls, irrespective of histological type and clinical stage [8]. Tumor stem cells can secrete immunosuppressive molecules, and further inhibit the killing effect of T cells, NK cells, and macrophages on tumor cells [9,10]. Therefore, targeting tumor stem cells can reduce the progression and recurrence of lymphoma.

Immunotherapy is the most promising treatment for malignant tumors, which has developed rapidly in recent years. As an immune checkpoint, cytotoxic T lymphocyte-associated antigen 4 (CTLA-4) plays the key role in tumor immunotherapy [11]. CTLA-4 is highly expressed on regulatory T (Treg) cells, which can bind to B7 molecules and inhibit the proliferation and activation of T cells. The expression of CTLA-4 in tumor stem cells was significantly higher than that in tumor cells [12]. Many studies have reported that CTLA-4 is highly expressed in multiple lymphoma types, including DLBCL, Hodgkin’s lymphoma, and follicular lymphoma [13,14]. However, its specific role and mechanism are not clear. The purpose of this study is to investigate the expression of CTLA-4 in DLBCL and to explore its correlation with tumor stem cells and Treg cells, so as to provide theoretical support for immunotherapy of lymphoma.

## 2 Materials and methods

### 2.1 Clinical data

Patients with primary DLBCL admitted to the Central Hospital of Yiyang city from October 2017 to October 2019 were included in this study. Inclusion criteria: age >18 years, clinical and pathological diagnosis was DLBCL. Exclusion criteria: other malignant tumors, autoimmune diseases, diabetes and uremia, AIDS, and viral hepatitis. According to the international prognosis index of lymphoma, patients were divided into low-risk group (score ≤2) and high-risk group (score >2). There were 20 patients in the low-risk group with an average age of 48.21 years, and 20 patients in the high-risk group with an average age of 51.26 years. Around 20 normal lymph nodes were selected as the control group. There were no significant statistical differences when the general data of the three groups were compared (Table 1).

**Table 1:** Clinicopathological data of the patients

Group	Control (*n* = 20)	Low-risk (*n* = 20)	High-risk (*n* = 20)
Sex (male)	15	14	16
Average age (years)	50.74	48.21	51.26
BMI (kg/m^2^)	19.17	18.93	18.29
Ki-67 (%)	4.23	32.74	59.98
CTLA-4 (AOD)	18.08	53.65	198.53
TGF-β (AOD)	16.55	28.63	59.13
CD44 (%)	0.09	1.02	1.73

AOD: average optical density.

**Informed consent:** Informed consent was obtained from all the individuals included in this study.**Ethical approval:** The research related to human use has complied with all relevant national regulations and institutional policies and is in accordance with the tenets of the Helsinki Declaration, and has been approved by the authors’ institutional review board or equivalent committee.

### 2.2 Histological staining

The patient’s lymphoma tissues were collected and fixed with 4% paraformaldehyde for 4 h. After paraffin sectioning, some sections were stained with hematoxylin-eosin (H&E; Sigma, USA) to detect basic pathological changes. Immunohistochemistry was performed to detect Ki-67 proliferation index, CTLA-4 expression, and CD44+ cell ratio (Abcam, UK).

### 2.3 Flow cytometry

Peripheral blood of the patients was collected and lymphocytes were isolated with lymphocyte separation medium (TBD, China). After washing with PBS three times, CD4-PE and CD25-FITC (BD, USA) were added and incubated in the dark for 15 min, then washed with PBS three times. After breaking cell membrane, Foxp3-APC (BD, USA) was added and incubated in the dark for 15 min. After washing with PBS three times, the proportion of CD4+ CD25+ Foxp3+ Treg cells in lymphocytes was detected by flow cytometry (BD, USA). Results were recorded on a lymphocyte population gated according to forward scatter and side scatter characteristics (FSC^low^/SSC^low^) [15].

### 2.4 Cell transfection

DLBCL cell line U2932 (ATCC, USA) was cultured in RPMI1640 medium containing 10% fetal bovine serum (Hyclone, USA). After washing with PBS three times, CTLA-4 plasmid and CTLA-4 siRNA (designed and synthesized by Shanghai Genechem Company, China) were transfected into U2932 using Lipofectamine 3000 (Thermo Fisher Scientific, USA). After 72 h of culture, U2932 was collected in each group. Expression of CTLA-4 was detected by flow cytometry, and TGF-β level was detected by ELISA.

### 2.5 Tumor stem cell detection

The experiments were divided into four groups: control group (U2932 was transfected with control siRNA), CTLA-4 inhibition group (U2932 was transfected with CTLA-4 siRNA), CTLA-4 group (U2932 was transfected with CTLA-4 plasmid), and TGF-β Neu group (U2932 was transfected with CTLA-4 plasmid, and then added TGF-β neutralization antibody). After 72 h of culture, U2932 was collected in each group. CD44-PE and CD34-APC (BD, USA) were added and incubated in the dark for 15 min. After washing with PBS three times, the proportion of CD34+ CD44+ cells was detected by flow cytometry (BD, USA).

### 2.6 MTT experiment

After 72 h of culture, 5 mg/mL MTT solution (Sigma, USA) was added in each group. After 4 h of culture, the supernatant was carefully discarded and 150 µL of DMSO (Sigma, USA) was added to each well. After shaking for 10 min, the absorbance values (OD: 570 nm) were measured on a microplate reader in each group.

### 2.7 Invasion experiment

We used Transwell invasive chamber (Corning, USA) to study the invasion of DLBCL cells. RPMI1640 containing 2 × 10^4^ CM-DiI (Sigma, USA) labeled U2932 was added to the upper chamber. RPMI1640 containing 10% fetal bovine serum was added to the lower chamber. After 12 h of culture, all cells in the lower chamber were collected, and flow cytometry was used for absolute cell counts.

### 2.8 Co-culture experiment

Peripheral blood of healthy volunteers was collected, and lymphocytes were isolated with lymphocyte separation medium. The experiments were divided into five groups: Control group (added the cell culture medium), lymphoma group (added U2932), CTLA-4 inhibition group (added U2932 transfected with CTLA-4 siRNA), CTLA-4 group (added U2932 transfected with CTLA-4 plasmid), and TGF-β Neu group (added U2932 transfected with CTLA-4 plasmid and TGF-β neutralizing antibodies). U2932 and lymphocytes were co-cultured in a 5% CO_2_ incubator. After 48 h of culture, cells in each group were collected. After washing with PBS three times, CD4-PE and CD25-FITC were added and incubated in the dark for 15 min, then washed with PBS three times. After breaking the cell membrane, Foxp3-APC was added and incubated in the dark for 15 min. The proportion of CD4+ CD25+ Foxp3+ Treg cells in lymphocytes was detected by flow cytometry after washing with PBS three times.

### 2.9 Animal experiment

For animal studies, 1 × 10^6^ U2932 cells in 30 mL PBS was slowly transplanted into the subcutaneous tissue of C57BL/6 mice. The experiments were divided into four groups: Control group (subcutaneous transplantation of U2932 cells), CTLA-4 inhibition group (subcutaneous transplantation of U2932 transfected with CTLA-4 siRNA), CTLA-4 group (subcutaneous transplantation of U2932 transfected with CTLA-4 plasmid), and TGF-β Neu group (subcutaneous transplantation of U2932 transfected with CTLA-4 plasmid, and tail vein injection of TGF-β neutralizing antibody). Two weeks after implantation, the mice were anesthetized with 0.1% pentobarbital sodium and then sacrificed. The transplanted tumors were obtained and fixed in 4% paraformaldehyde. After paraffin sectioning, some sections were stained with H&E. Immunohistochemistry was performed to detect the expression of Ki-67, CD44 and TGF-β (Abcam, UK).

**Ethical approval:** The research related to animal use has complied with all relevant national regulations and institutional policies for the care and use of animals, and were approved by the Ethics Committee of Central Hospital of Yiyang city, China.

### 2.10 Statistical method

Statistical analyses were performed using Graphpad Software 5.0. Data values were showed as mean ± SD. Multiple data comparisons were performed via analysis of variance and Bonferroni *post hoc* test. Correlation analyses were performed via Spearman’s rank correlation. *P* < 0.05 was considered statistically significant.

## 3 Results

### 3.1 CTLA-4 expression was significantly increased in DLBCL

**Figure 1:**
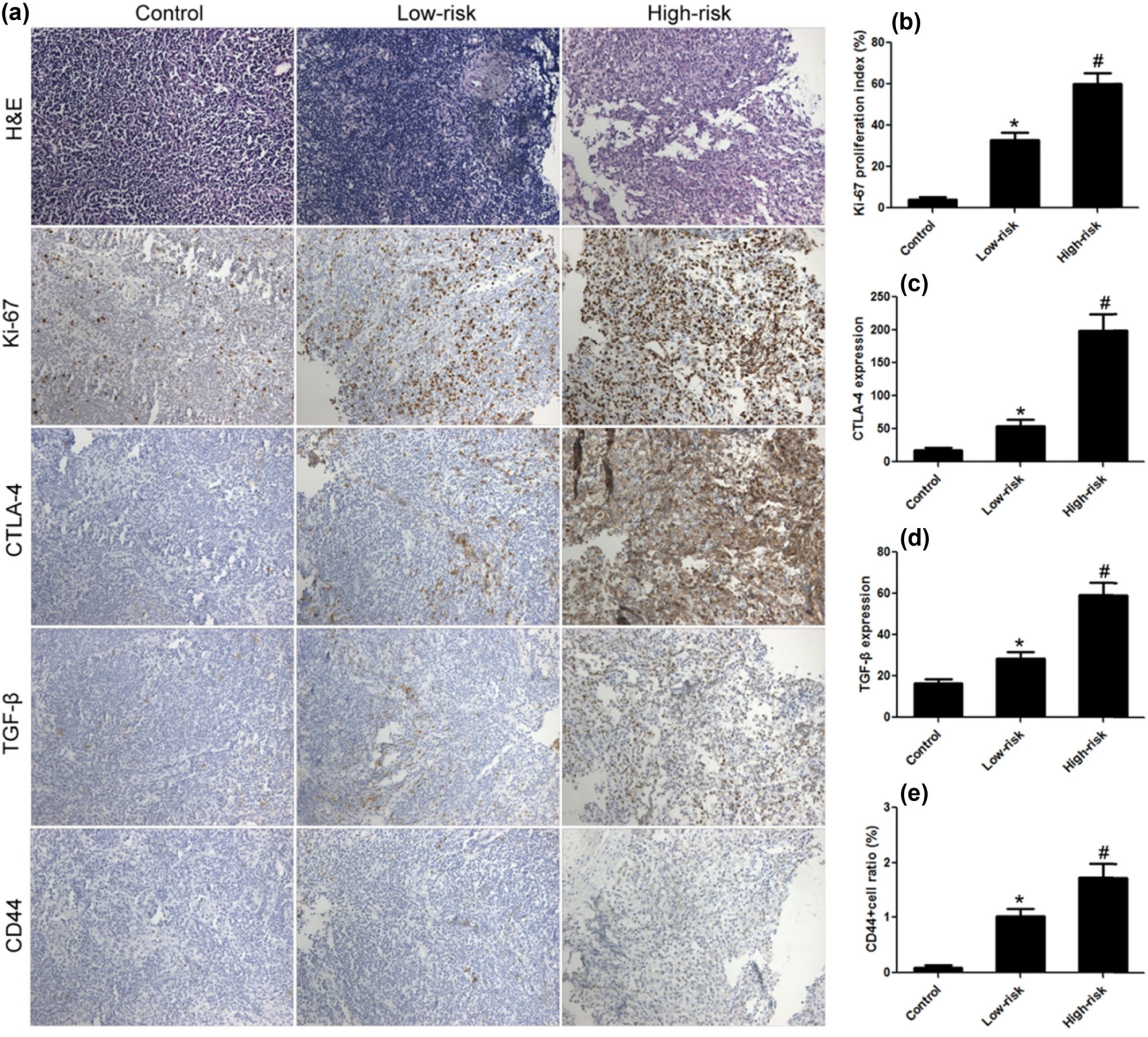
CTLA-4 expression was significantly increased in DLBCL. (a) H&E and immunohistochemical staining. Compared to the low-risk group, Ki-67 proliferation index (b), CTLA-4 (c), and TGF-β (d) expression, and CD44+ cell ratio (e) were significantly increased, respectively, in the high-risk group. *n* = 20, *VS Control, *P* < 0.05; ^#^ *VS* Low-risk, *P* < 0.05.

Compared to the control group, the Ki-67 proliferation index, CTLA-4 and TGF-β expression, and CD44+ cell proportion were significantly increased in the low-risk group. In the high-risk group, the Ki-67 proliferation index was 59.98%, the average optical density (AOD) of CTLA-4 and TGF-β was 198.52 and 59.13, and the proportion of CD44+ cells was 1.73%. The differences were statistically significant when compared with the low-risk group. Correlation analysis results showed that CTLA-4 expression was positively correlated with the proportion of CD44+ cells in lymphoma, indicating that CTLA-4 may have a certain correlation with tumor stem cells (Figure 1).

### 3.2 Proportion of Treg cells was significantly increased in DLBCL

**Figure 2:**
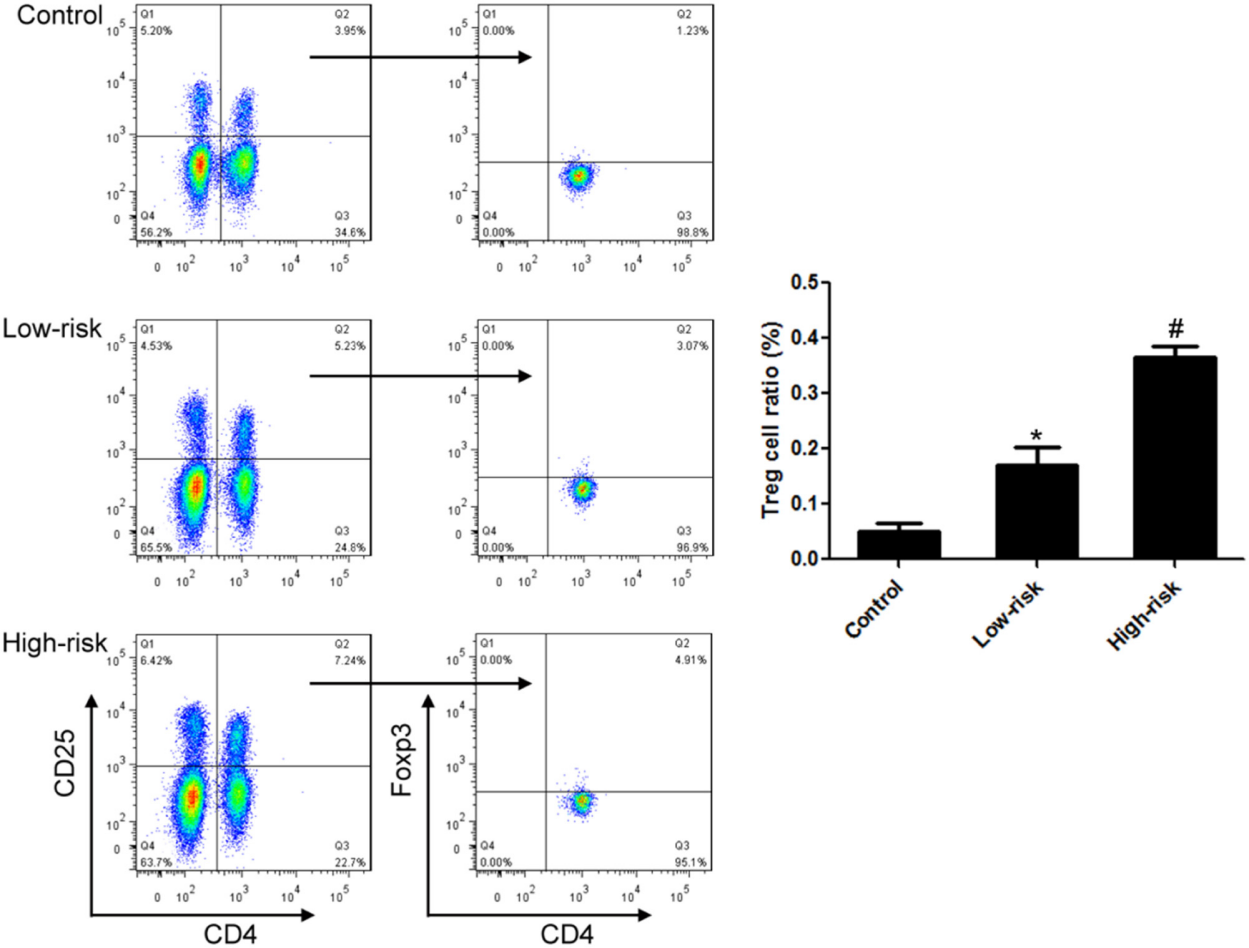
The proportion of Treg cells was significantly increased in DLBCL. Compared to the low-risk group, the proportion of Treg cells was significantly increased in the high-risk group. *n* = 20, *VS Control, *P* < 0.05; ^#^ *VS* Low-risk, *P* < 0.05.

Compared with the control group, the proportion of Treg cells in the low-risk group increased significantly. The proportion of Treg cells in the high-risk group was 0.37% compared to the low-risk group, and the difference was statistically significant. Correlation analysis showed that the expression of CTLA-4 was positively correlated with the proportion of Treg cells in lymphoma (Figure 2).

### 3.3 CTLA-4 increased TGF-β level in lymphoma

**Figure 3:**
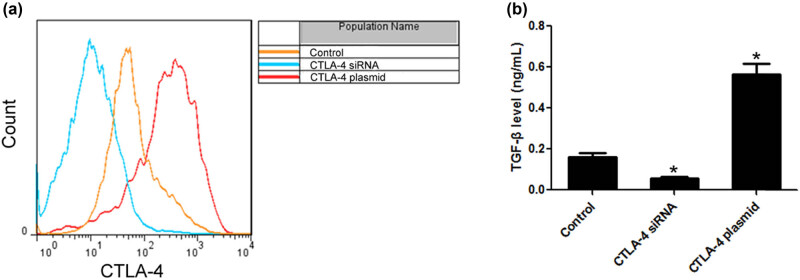
CTLA-4 activated TGF-β/Smad3 pathway in lymphoma. (a) Flow cytometry was used to detect CTLA-4 expression of lymphoma cells; (b) ELISA was used to detect TGF-β level in lymphoma cells. *n* = 6, *VS Control, *P* < 0.05.

Compared with the control group, the expression of CTLA-4 in the CTLA-4 plasmid group increased by 7.52 times, while that in the CTLA-4 siRNA group decreased by 83.33%. These indicated that both plasmid and siRNA were successfully transfected into lymphoma cells. ELISA results showed that CTLA-4 could significantly increase the level of TGF-β in lymphoma cell, while the level of TGF-β in CTLA-4 siRNA group was significantly decreased (Figure 3).

### 3.4 CTLA-4 increased the proportion of CD44+ CD34+ cells in lymphoma cells

**Figure 4:**
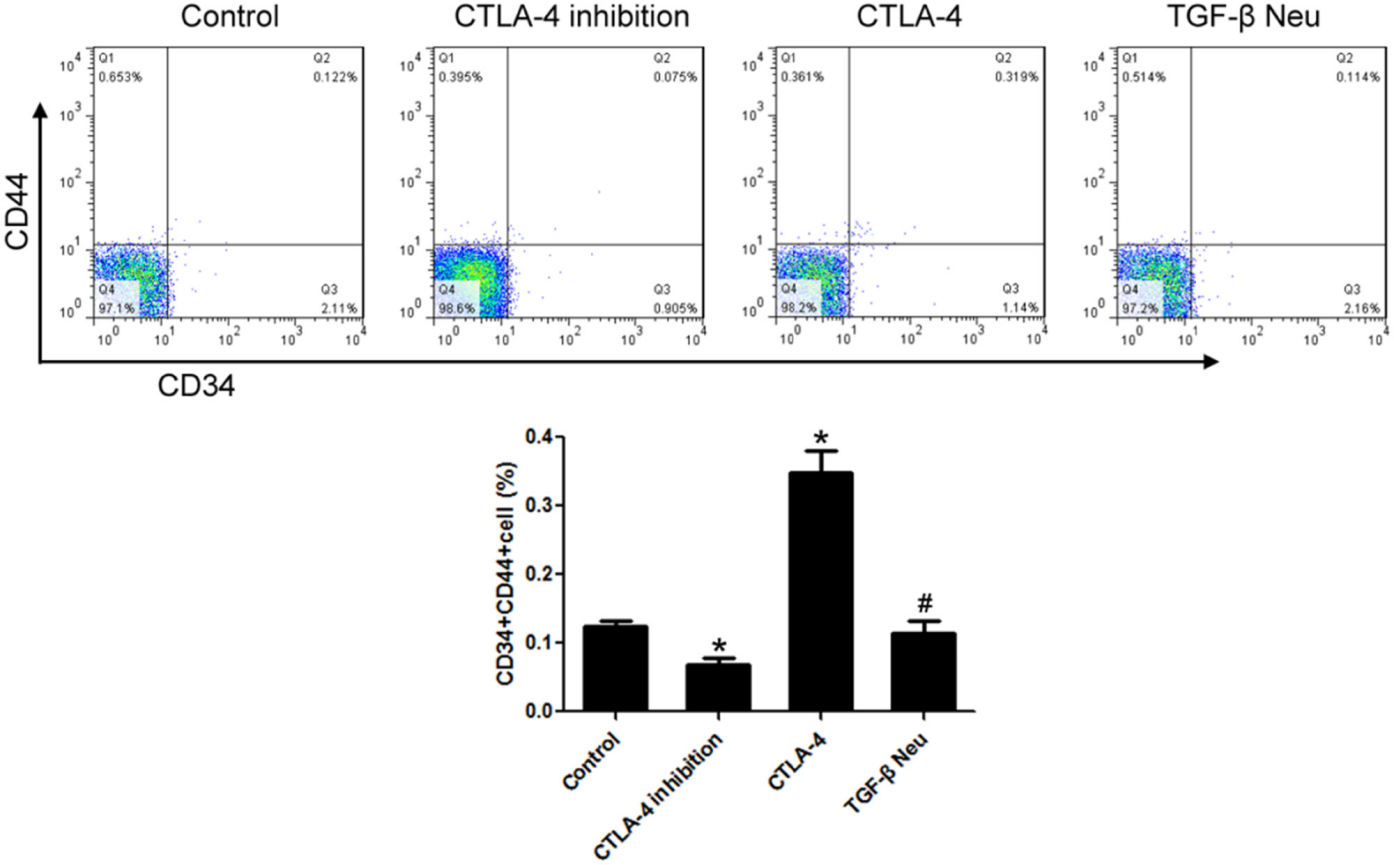
CTLA-4 increased the proportion of CD44+ CD34+ cells in lymphoma cells, and TGF-β neutralization could significantly block these effects of CTLA-4. Flow cytometry was used to detect the proportion of CD44+ CD34+ cells. *n* = 6, *VS Control, *P* < 0.05; ^#^ *VS* CTLA-4, *P* < 0.05.

Compared to the control group, the proportion of CD44+ CD34+ cells in the CTLA-4 inhibition group decreased significantly. CTLA-4 significantly increased the proportion of CD44+ CD34+ cells in lymphoma cells, and TGF-β neutralization could significantly block this effect of CTLA-4 (Figure 4).

### 3.5 CTLA-4 promoted the proliferation and invasion of lymphoma cells

**Figure 5:**
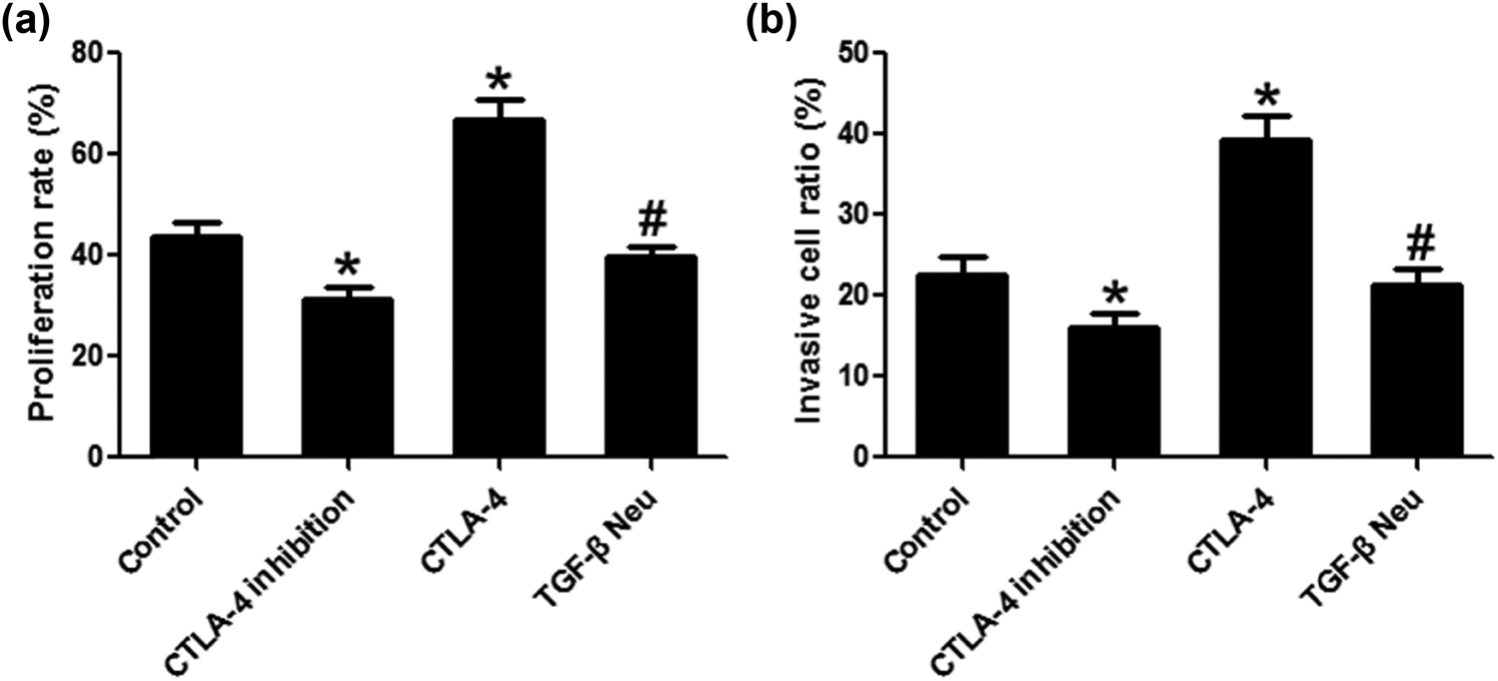
CTLA-4 promoted the proliferation and invasion of lymphoma cells. MTT experiment was used to detect lymphoma cell proliferation (a) and Transwell experiment was used to detect lymphoma cell invasion (b). *n* = 6, *VS Control, *P* < 0.05; ^#^ *VS* CTLA-4, *P* < 0.05.

Compared to the control group, the proliferation and invasion of lymphoma cells in the CTLA-4 inhibition group were significantly reduced. CTLA-4 significantly promoted the proliferation and invasion of lymphoma cells, and TGF-β neutralization can significantly block these effects of CTLA-4 (Figure 5).

### 3.6 Lymphoma increased the proportion of Treg cells in lymphocytes

**Figure 6:**
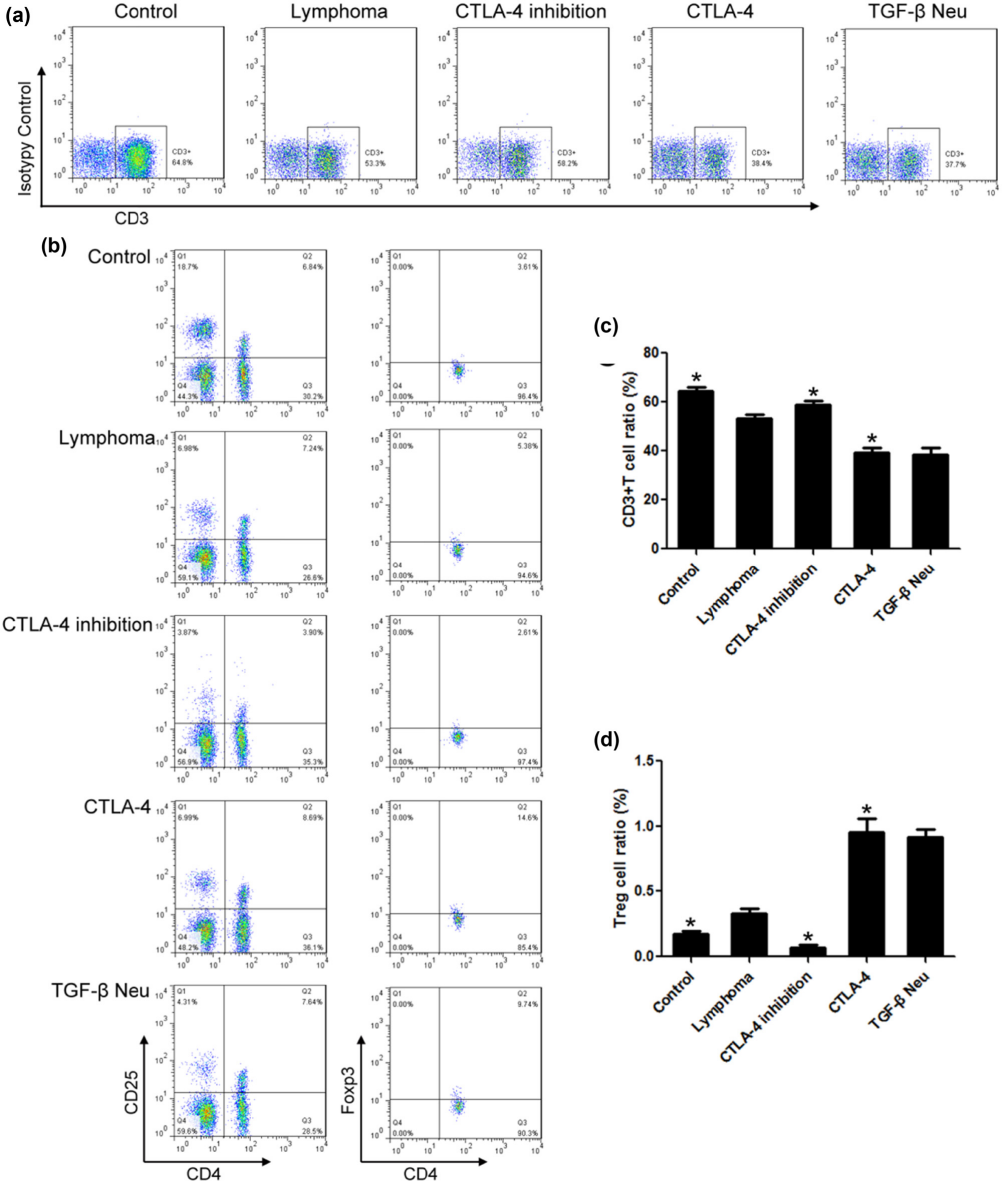
Lymphoma increased the proportion of Treg cells in lymphocytes. (a) Flow cytometry was used to detect the proportion of T cells. (b) Flow cytometry was used to detect the proportion of Treg cells. (c) Compared to the lymphoma group, the proportion of T cells decreased significantly in the CTLA-4 group. (d) Compared to the lymphoma group, the proportion of Treg cells increased significantly in the CTLA-4 group. *n* = 6, *VS Lymphoma, *P* < 0.05.

Compared to the control group, the proportion of Treg cells increased significantly in the lymphoma group, while the proportion of T cells decreased significantly. Compared to the lymphoma group, the proportion of Treg cells decreased significantly in the CTLA-4 inhibition group, while the proportion of T cells increased significantly. The proportion of Treg cells and T cells in the CTLA-4 group was 0.96 and 39.38%, and the differences were statistically significant when compared with the lymphoma group. TGF-β neutralization had no significant effect on the immunosuppression of CTLA-4 (Figure 6).

### 3.7 CTLA-4 promoted the growth and proliferation of transplanted lymphoma *in vivo*

**Figure 7:**
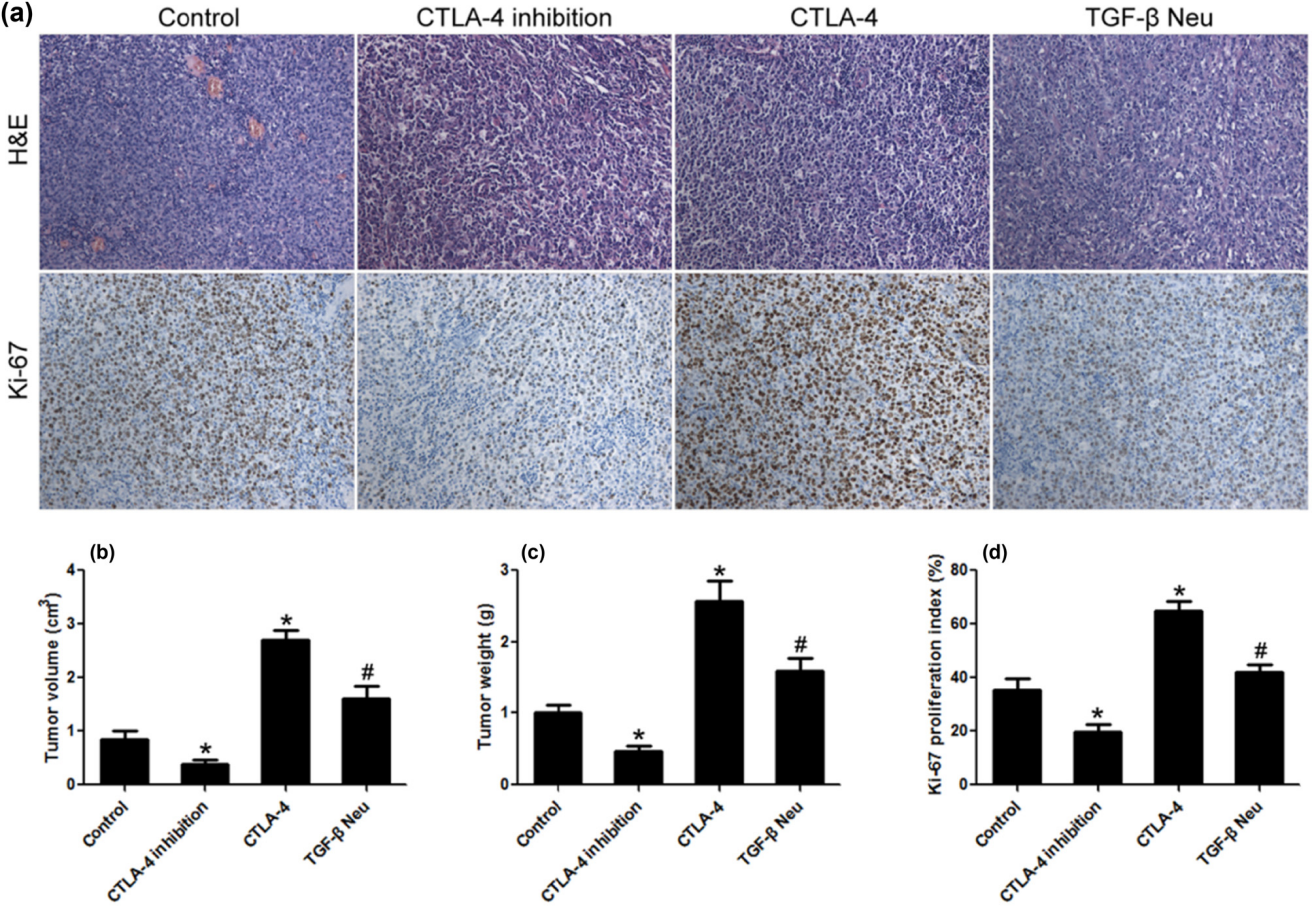
CTLA-4 promoted the growth and proliferation of transplanted lymphoma. (a) H&E and immunohistochemical staining; (b and c) CTLA-4 significantly increased the volume and weight of transplanted lymphoma; and (d) CTLA-4 promoted the proliferation of lymphoma cells *in vivo*. *n* = 10, *VS Control, *P* < 0.05; ^#^ *VS* CTLA-4, *P* < 0.05.

Compared to the control group, the volume and weight of the transplanted tumor were significantly less in the CTLA-4 inhibition group. CTLA-4 significantly increased the volume and weight of transplanted tumor, while TGF-β neutralization can partially inhibit these effects of CTLA-4. The results of immunohistochemistry showed that the Ki-67 proliferation index in the CTLA-4 inhibition group was significantly lower than that in the control group. CTLA-4 significantly increased the Ki-67 proliferation index, while TGF-β neutralization can partially inhibit this effect of CTLA-4 (Figure 7).

### 3.8 CTLA-4 increased the proportion of lymphoma stem cells *in vivo*

**Figure 8:**
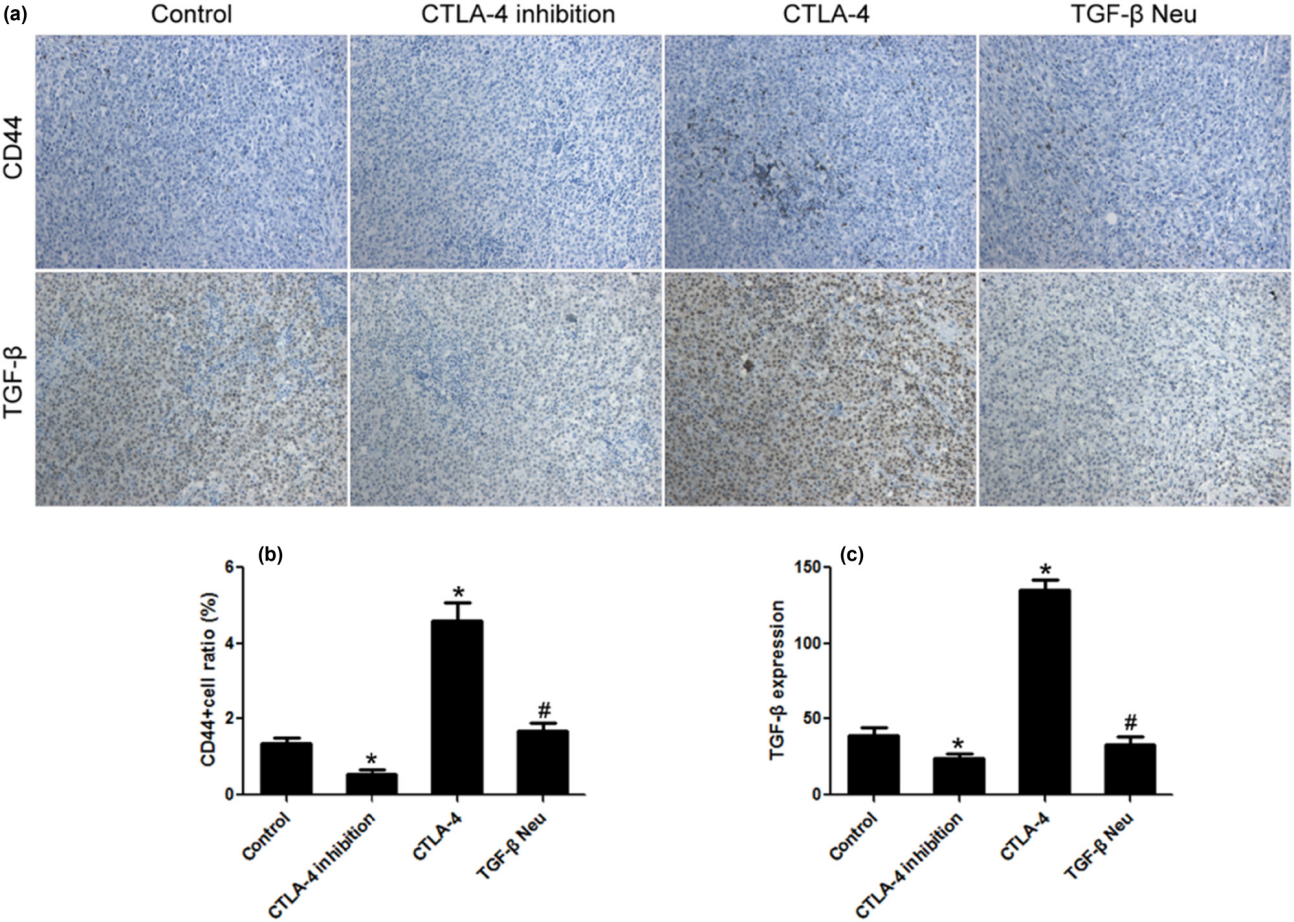
CTLA-4 increased the proportion of lymphoma stem cells *in vivo*. (a) Immunohistochemical staining; (b) CTLA-4 significantly increased the proportion of CD44+ cells in transplanted lymphoma; and (c) CTLA-4 enhanced the expression of TGF-β *in vivo*. *n* = 10, *VS Control, *P* < 0.05; ^#^ *VS* CTLA-4, *P* < 0.05.

When compared with the lymphoma group, the proportion of CD44+ cells and the expression of TGF-β in the CTLA-4 inhibition group decreased significantly. CTLA-4 significantly increased the proportion of CD44+ cells and the expression of TGF-β. TGF-β neutralization could completely inhibit these effects of CTLA-4 (Figure 8).

## 4 Discussion

Malignant tumors are the leading cause of death worldwide, and immunotherapy is considered the most promising treatment. Targeting the immune checkpoint can significantly inhibit the progression and metastasis of cancer, and several targeted drugs have been used in clinical studies [16]. CTLA-4 is one of the most important immune checkpoints, which is highly expressed in regulatory immune cells and inhibits the proliferation and activation of T cells [17]. Clinical studies showed that the CTLA-4 inhibitor could significantly prolong the survival time of patients with malignant tumors [16]. Many studies have shown that CTLA-4 is highly expressed in cancer cells and can be used for early diagnosis, treatment monitoring, and prognosis prediction of cancer [18]. Our study found that compared to normal lymphoid tissue, CTLA-4 expression in lymphoma tissues was significantly increased and was closely related to the malignant degree of lymphoma, indicating that CTLA-4 could be used as an indicator for early diagnosis and clinical treatment of lymphoma.

Treg cells are the most important regulatory immune cells and they mainly suppress cellular immunity [19]. In addition, Treg cells also secrete many immunosuppressive molecules such as IL-10, which can inhibit the immune response mediated by T cells, B cells, and macrophages. Clinical studies have proved that Treg cells increased in cancer patients, and its number is closely related to disease condition and prognosis [20,21]. Our results showed that the proportion of Treg cells in the high-risk group was significantly higher than that in the low-risk group. Lymphoma cells with higher CTLA-4 expression can induce the proliferation of Treg cells, which can recruit more immunosuppressive cells into the tumor and suppress the anti-tumor immune response.

Tumor stem cells are closely related to tumor progression, metastasis, and recurrence [22]. Tumor stem cells are not sensitive to chemoradiotherapy and can be the root of tumor recurrence and metastasis [23]. Therefore, finding new targets to kill tumor stem cells has important clinical significance. Recent studies have found that lymphoma stem cells play an important role in immune escape, drug resistance, and recurrence of lymphoma [24]. Our study found that the number of tumor stem cells in the high-risk group was significantly higher than that in the low-risk group, and CTLA-4 was highly correlated with tumor stem cells. CTLA-4 can increase the proportion of lymphoma stem cells and promote the proliferation and invasion of lymphoma cells. The results of animal experiments further confirmed that CTLA-4 could promote the growth of transplanted tumors.

TGF-β plays an important role in embryonic development, tissue repair, and stem cell proliferation [25]. Similar to CTLA-4, TGF-β also has a strong immunosuppressive function [26]. Moreover, many studies have showed that TGF-β is an important signaling pathway for tumor stem cell formation [27]. TGF-β can promote epithelial–mesenchymal transition in breast cancer cells. Some cancer cells begin to express stem cell markers, such as CD44 and CD34, and their self-renewal, proliferation, and drug resistance are significantly increased [28]. Our study found that CTLA-4 can increase the number of lymphoma stem cells and enhance the proliferation and invasion ability of lymphoma cells through the TGF-β pathway. Animal studies also confirmed these results, and TGF-β blocking significantly inhibited these effects of CTLA-4.

In summary, CTLA-4 promoted lymphoma progression by increasing the number of tumor stem cells and Treg cells. This study provided theoretical support for the immunotherapy of lymphoma.
